# The effect of progesterone replacement on gene expression in the corpus luteum during induced regression and late luteal phase in the bonnet monkey (*Macaca radiata*)

**DOI:** 10.1186/1477-7827-9-20

**Published:** 2011-02-03

**Authors:** Padmanaban S Suresh, Kadthur C Jayachandra, Rudraiah Medhamurthy

**Affiliations:** 1 Department of Molecular Reproduction, Development and Genetics, Indian Institute of Science, Bangalore-560012, India

## Abstract

**Background:**

In higher primates, although LH/CG play a critical role in the control of corpus luteum (CL) function, the direct effects of progesterone (P4) in the maintenance of CL structure and function are unclear. Several experiments were conducted in the bonnet monkey to examine direct effects of P4 on gene expression changes in the CL, during induced luteolysis and the late luteal phase of natural cycles.

**Methods:**

To identify differentially expressed genes encoding PR, PR binding factors, cofactors and PR downstream signaling target genes, the genome-wide analysis data generated in CL of monkeys after LH/P_4 _depletion and LH replacement were mined and validated by real-time RT-PCR analysis. Initially, expression of these P4 related genes were determined in CL during different stages of luteal phase. The recently reported model system of induced luteolysis, yet capable of responsive to tropic support, afforded an ideal situation to examine direct effects of P4 on structure and function of CL. For this purpose, P4 was infused via ALZET pumps into monkeys 24 h after LH/P4 depletion to maintain mid luteal phase circulating P4 concentration (P4 replacement). In another experiment, exogenous P4 was supplemented during late luteal phase to mimic early pregnancy.

**Results:**

Based on the published microarray data, 45 genes were identified to be commonly regulated by LH and P4. From these 19 genes belonging to PR signaling were selected to determine their expression in LH/P_4 _depletion and P4 replacement experiments. These 19 genes when analyzed revealed 8 genes to be directly responsive to P4, whereas the other genes to be regulated by both LH and P4. Progesterone supplementation for 24 h during the late luteal phase also showed changes in expression of 17 out of 19 genes examined.

**Conclusion:**

These results taken together suggest that P4 regulates, directly or indirectly, expression of a number of genes involved in the CL structure and function.

## Background

In mammals, the secretion of progesterone (P_4_) by corpus luteum (CL) is absolutely essential for establishment and, in some species, maintenance of pregnancy. In higher primates, LH and chorionic gonadotropin (CG) have been suggested to be the principal trophic factors responsible for P_4 _secretion in the CL [1]. Whether P_4 _plays a role in the maintenance of structure and function of CL has not been fully elucidated in higher primates. Rothchild postulated that P_4 _is the primary stimulus of its own secretion and that intraluteal P_4_, among other effects such as control of structural integrity and steroidogenic capacity, is responsible for regulation of production of luteolysin, the prostaglandin (PG) F_2α_, within the CL[2,3]. More recent studies have provided several lines of evidence, some of them with mechanistic insights, in support of the direct effects of P_4 _on CL. Expression of progesterone receptor (PR) isoforms in CL have been reported in several mammalian species [4-6]. Several studies have suggested that by way of its proliferative and anti-apoptotic actions, P_4 _functions as survival factor of CL in human [7], rat [8-10], and cattle [11,12]. Like other steroid nuclear receptors, PR utilizes a plethora of cofactors termed coactivators or corepressors to regulate gene expression [13]. The PR cofactors identified to date include coactivators like (SRC1-3, CBP/p300 and NCOA1-3) [13] and corepressors like NCOR1-2 involved in modulation of PR activity in vivo [14,15].

We have recently standardized a GnRH R antagonist-induced luteolysis model system in the monkey in which induced luteolysis could be reversed by exogenous LH administration [16]. Employing this model system, microarray analysis of differentially expressed genes in CL tissue during induced luteolysis and LH replacement following induced luteolysis has been determined [16]. The GnRH R antagonist-induced regressed CL with ablated LH action, yet capable of responding to LH replacement, affords an ideal situation to examine direct effects of P_4 _on CL structure and function. Extensive tissue remodeling with breakdown and renewal of extracellular matrix (ECM) that occurs during spontaneous luteolysis requires participation of matrix metalloproteinases (MMPs) and their tissue inhibitors, TIMPs [17-19]. It has been reported that decrease in P_4 _levels during late luteal phase of the non fertile cycle is associated with changes in expression of ECM regulators [20]. It remains to be determined whether P_4 _regulates expression of tissue proteinases, especially following conception. The purpose of this study was to examine effects of P_4 _action on gene expression changes and function of CL. Experiments were carried out to determine expression of genes in CL tissue that encode different elements of PR complex and few downstream targets of PR activation throughout the luteal phase, after LH/P_4 _depletion, after P_4 _replacement and after P_4 _supplementation during the late luteal phase.

## Methods

### Reagents

Oligonucleotide primers were synthesized by Sigma-Genosys, Bangalore, India. DyNAzyme™ II DNA polymerase (F-501L) was purchased from Finnzymes, Espoo, Finland. Moloney murine leukemia virus (MMuLV) reverse transcriptase (RT) and 100 bp DNA ladder were obtained from MBI Fermentas GmbH (St. Leon-Rot, Germany). Power SYBR^® ^Green PCR master mix was obtained from Applied Biosystems, Foster City, CA, USA. GnRH R antagonist [Cetrorelix^®^; (CET)] was a kind gift from Asta Medica, Frankfurt, Germany. ALZET^® ^Osmotic pump Model 2ML1 (infusion rate 10 μl/h) was obtained from Alza Corporation, Palo Alto, CA. Antibodies specific to phospho-p38 (9211), phospho-p42/44 MAPK (9101), p38 MAPK (9212), p42/44 MAPK (9102), pMKK3/6 (9231), p38 MAPK assay kit (9820), MMP-9 (G657) and Phototype-HRP Western detection system with horseradish peroxidase-linked anti-rabbit IgG (7071) were purchased from Cell Signaling Technology, Inc. Danvers, MA. Antibodies specific to PR (sc-538), NCOA1 (sc-8995), NCOA2 (also designated as GRIP1; sc-8996), NCOA3 (sc-25742) were procured from Santa Cruz Biotechnology, Santa Cruz, CA. Crystalline P_4 _(P0130) and all other reagents were purchased from Sigma Aldrich Corp. St. Louis, MO or sourced locally.

### Animal protocols, blood samples and CL collection

Experimental protocols involving monkeys in this study were approved by the Institutional Animal Ethics Committee of the Indian Institute of Science, Bangalore. Adult female bonnet monkeys (*Macaca radiata*) weighing 3.3-5.1 kg were utilized for the study. The general care and housing of monkeys have been described elsewhere [21]. In this study, one day after occurrence of peak E_2 _surge was designated as day 1 of the luteal phase, and CL was collected on designated days of the luteal phase and/or after administration of different treatments (see below). To retrieve CL from experimental monkeys, ovaries were accessed by performing laparotomy under aseptic conditions on ketamine hydrochloride (15 mg/kg BW) and/or pentobarbital sodium (8-12 mg/kg BW) anesthetized monkeys. Under sterile conditions, the excised CL was transferred to a petri dish containing filter paper, wiped dry, weighed, cut into 4-5 pieces, placed in individual sterile cryovials, snap-frozen in liquid nitrogen and stored at -70°C until analysis.

### Experiment 1: Examination of differentially expressed genes during induced luteolysis and LH replacement experiments

In order to identify genes associated with LH and P_4 _receptor signaling, the microarray data of induced luteolysis (LH/P_4 _depletion) and rescue of CL function by exogenous LH replacement studies deposited (#GSE7827 and #GSE8371) by us previously were examined. The main focus was on identifying changes in expression of genes associated with PR and genes downstream of PR signaling. For this purpose, various cofactors and corepressors (NCOA1-3, NCOR1-2, FKBP4 and FKBP5) of PR were selected for analysis. Expression of MMP-2 and 9, TIMP-3, ADAMTS-1, PGRMC1, BMP5, CDH2, WNT7A, HOXA1, IHH genes regarded as downstream target of gonadotropin and/or PR signaling in CL and in traditional P_4 _target tissues such as uterus were also examined [19,22-26]. The expanded forms of gene symbols used in this study are provided in Table 1. Real time RT-PCR analysis was carried out on RNA samples isolated from CL tissues collected during luteolysis [VEH (n = 5 animals) and LH/P_4 _depletion (n = 5 animals)] and LH rescue (LH replacement, n = 3 animals each for PBS and LH treatments) of CL function experiments as reported previously [16].

**Table 1:** List of expanded forms of gene symbols used

**Sl. No**.	Gene symbol	Gene name
1	PR	Progesterone receptor
2	PGRMC1	Progesterone receptor membrane component 1
3	FKBP4	FK506 binding protein 4
4	FKBP5	FK506 binding protein 5/peptidyl-prolyl cis-trans isomerase FKBP5-like (*M. mulatta*)
5	NCOA1	Nuclear receptor coactivator 1
6	NCOA2	Nuclear receptor coactivator 2
7	NCOA3	Nuclear receptor coactivator 3
8	NCOR1	Nuclear receptor corepressor 1
9	NCOR2	Nuclear receptor corepressor 2
10	WNT7A	Wingless-type MMTV integration site family, member 7A
11	AREG	Amphiregulin
12	BMP5	Bone morphogenetic protein 5
13	CDH2	Cadherin 2, type 1, N-cadherin (neuronal)/cadherin-2-like (*M. mulatta*)
14	HOXA1	Homeobox A1
15	IHH	Indian hedgehog
16	ADAMTS1	A disintegrin-like and metallopeptidase with thrombospondin type 1 motif, 1/ADAM metallopeptidase with thrombospondin type 1 motif, 1
17	MMP9	Matrix metallopeptidase 9
18	MMP2	Matrix metallopeptidase 2
19	TIMP3	Tissue inhibitor of metalloproteinase 3/TIMP metallopeptidase inhibitor 3 (*M. mulatta*)

### Experiment 2: Expression of various components of PR signaling complex and PR target genes throughout the luteal phase

To study expression of PR, various components of PR signaling complex and some of the downstream target genes of PR signaling, corpora lutea (n = 3 animals/stage of luteal phase) were collected from monkeys at early (day 5), mid (day 8), and late (day 14) stage of the luteal phase of the menstrual cycle as reported previously [27]. Also, CL (n = 3 animals) was collected from monkeys on day 1 of menstrual cycle (d1M), a time point when luteolytic events are manifested. Blood samples were collected from monkeys on the day of CL collection for determination of P_4 _concentration.

### Experiment 3: Effects of P_4 _replacement on expression of PR, coactivators, corepressors and P_4 _target genes during induced luteolysis

We have recently reported that replacement of LH post LH/P_4 _depletion leads to brisk and sustained increase in P_4 _concentration suggesting rescue of CL function following reestablishment of LH levels [16]. In the present experiments, we determined the direct effects of P_4 _on CL during absence of luteotrophic stimulus. The different components of the PR signaling complex and expression of few genes considered as target of PR activation were examined. In experiment 3.1, with a view to mimic mid luteal phase circulating P_4 _concentration, exogenous P_4 _was administered through implantation of P_4 _filled ALZET pumps to monkeys depleted of LH/P_4 _(P_4 _replacement model). For this purpose, pilot experiments were carried out in adult female monkeys to examine the feasibility of providing P_4 _as continuous infusion by way of implantation of P_4 _filled ALZET pump. Keeping in mind the limitation on use of organic solvents compatible with the pump, initially 16 mg of P_4 _was dissolved in 300 μl of ethanol and the solution was made up to 2 ml by propylene glycol and the entire solution was transferred to 2 ML1 ALZET pump. It was determined that three, 32.5 mg of P_4 _filled pumps that provided infusion of 487.5 μg/30 μl/h, were required to be implanted in anesthetized monkeys to establish circulating P_4 _concentration higher or in the range of mid luteal phase concentration. In order to determine the time course of P_4 _secretion immediately before and at different time intervals after injection of CET (LH/P_4 _depletion), monkeys on day 7 of the luteal phase (n = 3 animals) were administered CET (150 μg/kg BW, s.c.) and blood samples were collected twice daily until onset of menses. In experiment 3.2, three groups of monkeys (n = 3-4 animals/group) were administered 5.25% glucose (vehicle for CET treatment; VEH; group 1), CET [150 μg/kg BW; (LH/P_4 _depletion); CET; group 2] and CET (150 μg/kg BW) followed 24 h later with implantation of P_4 _filled three ALZET pumps designed to infuse 487.5 μg of P_4_/30 μl/h for 24 h [CET+P_4_; (P_4 _replacement); group 3]. Blood samples were collected immediately before and at different time intervals throughout the experiment. In group 3, ALZET pumps were removed 24 h later, and further blood samples were collected to monitor circulating P_4 _levels. Corpora lutea were retrieved from monkeys of all three groups (VEH, CET and CET+P_4_).

### Experiment 4: Effects of P_4 _supplementation on CL function during late luteal phase

An experiment was conducted to gain insight into direct effects of increased P_4 _during rescue of CL function that occurs during late luteal phase of the fertile menstrual cycle. On day 13 of the luteal phase of non-mated females, three P_4 _filled ALZET pumps (97.5 mg of P_4_) were implanted for 24 h, CL (n = 3 animals) was harvested and the pumps were removed 24 h later. Blood samples were collected immediately before and at different time intervals during implantation and after retrieval of CL as well as removal of implants for determining the P_4 _secretion pattern. For purposes of comparison, CL (n = 3 animals) was harvested from untreated monkeys (untreated control) on day 14 of the luteal phase of the menstrual cycle.

### RNA isolation

Total RNA was isolated from CL tissues obtained from different experiments using TRI reagent according to manufacturer's instructions. RNA samples were analyzed using NanoDrop ND-1000 UV-VIS spectrophotometer and samples with A_260_/A_280 _values ~1.8 -1.9 were selected for further analyses.

### Real-time RT-PCR analysis

Real time RT-PCR analysis was carried out as described previously [16]. Total RNA (1 μg) extracted from CL of monkeys from various experiments were reverse transcribed using MMuLV RT in Eppendorf mastercycler, epgradient PCR machine. Briefly, In a 20 μl total reaction, 1 μg of total RNA along with 1 μl of Oligo dT primer were incubated at 65°C for 10 min and then snap chilled on ice for 5 min. To this RNA-Oligo dT hybrid, a cocktail containing 4 μl of 5X RT buffer [250 mM Tris HCl (pH 8.3 at 25°C), 250 mM KCl, 20 mM MgCl_2 _and 10 mM DTT], 10 mM dNTPs and DEPC treated water were added followed by addition of 200 units (1 μl) of Revert Aid™MMuLV RT enzyme. The reaction mixture was incubated at 42°C for 60 min. The resulting cDNAs were used as template for real time RT-PCR analysis using gene specific primers. The primer pairs were designed using Rhesus macaque sequences submitted at NCBI and ENSEMBL using Primer Express software v2.0 (Applied Biosystems, Foster City, CA, USA) spanning the exon-exon junction. The diluted cDNA samples equivalent to 10 ng of total RNA were subjected to analysis in Applied Biosystems 7500 Fast Real Time PCR system with SDS v 1.4 program employing Power SYBR green 2X PCR master mix. The 10 μl real time RT-PCR mixture contained cDNA equivalent to 10 ng of total RNA, 5 μl of PCR master mix and 5 μM each of forward and reverse gene specific primers. The initial denaturation was carried out at 95°C for 10 min, with further 40 cycles of denaturation (95°C for 30 sec), annealing (specific annealing temperature for 30 sec) and extension (72°C for 30 sec) with a final extension of 5 min at 72°C. PCR reactions were carried out in duplicates in 96 well plates. For each gene a no template control (NTC) was included and dissociation/melting curves were generated to determine the specificity of primers. Real time RT-PCR data for each gene was normalized using L19 expression level as internal control within each cDNA sample. The fold change in expression of the genes was determined using the ΔΔC_t _method, which calculates the fold change using the formula: Fold change = 2^-ΔΔCt^, where C_t_= Threshold cycle i.e. the cycle number at which the relative fluorescence of test samples increases above the background fluorescence and ΔΔC_t_= [C_t _gene of interest (treated sample)-C_t _of L19 (treated sample)] - [C_t _gene of interest (control sample)-C_t _of L19 (control sample)]. The list of genes and details of the primers employed in the real time RT-PCR analysis along with the annealing temperature and expected product size are provided in Table 2.

**Table 2:** List of Primers used for real time RT-PCR analysis

**S. No**.	Gene name	Primer sequence (5'to 3')	Annealing temp (OC)	Product size (bp)
1	PR F	GCCACATTCAACACCCACTT	57.4	134
	PR R	CCTTCAGCTCAGTCATGACG		
2	NCOA1 F	CTGCACGTGGGGGATCAT	60.4	146
	NCOA1 R	CCTGGCTCATCTGGAGGGT		
3	NCOA2 F	CATGACCTCAGTGACCTCCGT	60.4	116
	NCOA2 R	CCATTCCAGGCAGCTGGTTT		
4	NCOA3 F	CACCACAGGGCAGATGAGTG	60.4	135
	NCOA3 R	TGTGGGGGGCTACTCATG		
5	NCOR1 F	GCATCGAGCTGCTGTTATCCC	62.9	145
	NCOR1 R	TCTCTGCCGCTGCTCCTCC		
6	NCOR2 F	AAGCAGCGAGCGGCTGCCAT	72.6	143
	NCOR2 R	TGCTGAGGGGCGTCGCTCTC		
7	FKBP4 F	GGGGACCGAGTCTTTGTCCACT	69.9	124
	FKBP4 R	CCCAAGCCTTGATGACCTCCC		
8	MMP-2 F	GCCACATTCTGGCCTGAGCT	62.4	151
	MMP-2 R	CCAGGCTGGTCAGTGGCTTG		
9	MMP-9 F	CTGGAGGTTCGACGTGAAG	55	155
	MMP-9 R	AACTCACGCGCCAGTAGAAG		
10	TIMP3 F	CAAGTACCAGTACCTGCTGACA	55.4	122
	TIMP3 R	GATAGTTCAGCCCCTTGC		
11	BMP5 F	CCTGAAGAGTCGGAGTACTCAG	59.2	139
	BMP5 R	GACTCTGGGTGGTCAGAGGA		
12	CDH2 F	CATCCCTCCAATCAACTTGCC	59.2	131
	CDH2 R	GAGGCTGGTCAGCTCCTG		
13	HOXA1 F	CTTCGCAGGACCAGGTCACTC	65.1	124
	HOXA1 R	GGTCCGAGGGGTAGGCTCG		
14	IHH F	GCCGCGCGGTGGACATCA	71.9	126
	IHH R	CGGAGCAATGCACGTGGGCC		
15	FKBP5 F	GGTGAAGCCCAGCTGCTC	66.7	114
	FKBP5 R	CTGGCACATGGAGATCTGC		
16	PRMC1F	CGCCGCTCAACCTGCTGCT	69.7	139
	PRMC1 R	GTGAAGTCGCGCCGCTTGAG		
17	ADAMTS 1 F	GCCGACTGGGAAAGCGGA	66.6	120
	ADAMTS 1 R	CCAGTTCCTGTGGGCTGTCC		
18	WNT7A F	AGCTGGGCTACGTGCTCAAGG	68.2	138
	WNT7A R	CCAGGTCCGTGTCCATGGG		
19	AREG F	GTTGCCCCAGAGACCGAGACG	72.6	113
	AREG R	CAGCATAATGGCCTGAGCCGA		
20	L19 F	GCCAACTCCCGTCAGCAGA	60	154
	L19 R	TGGCTGTACCCTTCCGCTT		

### Hormone assays

E_2 _and P_4 _concentrations in serum were determined by specific RIA as reported previously [21].

### Immunoblotting analysis

Immunoblot analyses and in vitro P38MAPK (P38) assays of CL tissue lysates were carried out as per published procedures. Assays were carried out with equal amount of protein lysates from the luteal tissue of various treatments as reported previously from the laboratory [27].

### Statistical analysis

Data were expressed as mean ± SEM. Statistical evaluation of mean differences of serum P_4 _concentrations and real-time RT-PCR expression among different experimental groups were analyzed by one-way ANOVA, followed by the Newman-Keuls multiple comparison tests (PRISM GraphPad, version 4.0; GraphPad Software Inc., San Diego, CA) and Student's t- test to compare between two groups. A P value of <0.05 was considered statistically significant. The linear regression analysis on microarray and real time RT-PCR data was done as reported earlier [16].

## Results

### Identification and validation of differentially expressed genes in LH/P_4 _depletion and LH replacement experiments

The experimental details of CL collection from LH/P_4 _depletion (CET-induced luteolysis) and LH replacement (CET+LH) models and the microarray data comparing these models deposited in NCBI's Gene Expression Ominibus (#GSE7827 and #GSE8371) have been described previously [16]. The published microarray data was mined for P_4← _responsive genes, and 45 differentially expressed genes were identified based on criteria of >1.5 fold change in both LH/P_4 _depletion and LH replacement experiments. We narrowed down to the 45 genes by comparing our previous data on LH replacement studies with P_4 -_responsive genes reported in literature for other tissues [28,29]. From the 45 genes identified, 19 genes belonging to steroidogenesis and P_4 _target/regulation related genes (PR, PR binding proteins, cofactors, corepressors and some of the genes considered as downstream targets of PR activation) considered necessary for CL structure and function were selected for further studies. It should be noted that of the 19 genes, expression of only 12 genes was examined during different stages of the luteal phase in experiment 2 and expression of all 19 genes was determined in experiments 3 and 4.

**Figure 1:**
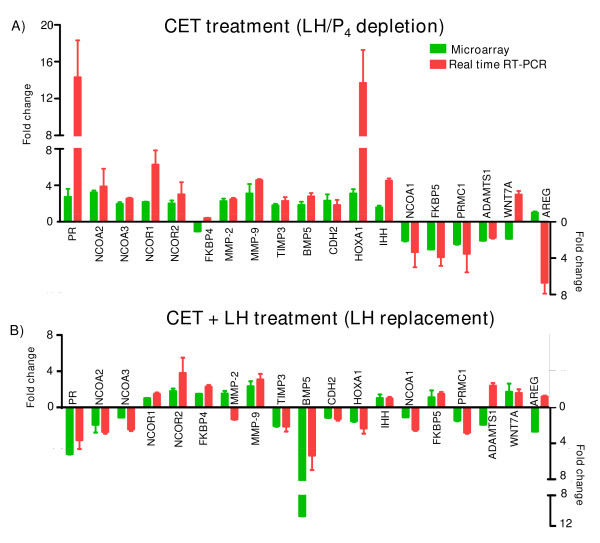
**Microarray and real time RT-PCR expression analyses of genes associated with PR signaling/regulation in CL tissue from monkeys of LH/P_4← _depletion and LH replacement models**. (A) Effects of LH/P_4 _depletion (CET treatment; n = 5 animals each for VEH and CET treatments) and (B) LH replacement (CET+LH treatments; n = 3 animals each for CET+PBS and CET+LH treatments) on gene expression changes in the CL tissue. Microarray data deposited at GEO with accession #s GSE7827 and GSE8371 were analyzed. Expression of P_4 _responsive genes identified from the microarray data (i.e. having >1.5 fold cut off over VEH treatment) is expressed as fold expression change (mean ± SEM) and further validated by real time RT-PCR analyses. The real time RT-PCR analysis was determined using ΔΔC_t _method (see materials and methods). RT-PCR and microarray data are presented mean ± SEM of fold change above control.

Figure 1A shows microarray and real time RT-PCR fold changes in expression of genes associated with PR signaling/regulation in CL tissue from monkeys of LH/P_4← _depletion and LH replacement models. Validation of microarray data from LH/P_4← _depletion model by real time RT-PCR analyses provided a good correlation in expression changes of most genes examined except for PR, NCOR1, and HOXA1 which showed higher expression changes in real time RT-PCR analysis, while expression of WNT7A and AREG genes showed inverse correlation by real time RT-PCR analysis (Figure 1A). The linear regression analysis on log_2_-transformed data of microarray and real time RT-PCR analyses suggested a good correlation (R = 0.5013, P = 0.0484). A number of genes that were examined to be differentially expressed after LH/P_4← _depletion were found to be reversed following LH replacement (Figure 1B). Expression of P_4← _responsive genes like PR, NCOA3, TIMP3, BMP5 and CDH2 was down regulated after LH replacement, whereas expression of genes like NCOR1, NCOR2 and MMP-9 was up regulated after LH replacement (Figure 1B). The microarray data and real time RT-PCR data for LH replacement experiments showed good correlation except for the observation of profound down regulation in BMP5 expression by microarray analysis than real time RT-PCR analysis (R = 0.7028, P = 0.0312).

### Expression of PR cofactors and its downstream target genes throughout the luteal phase

**Figure 2:**
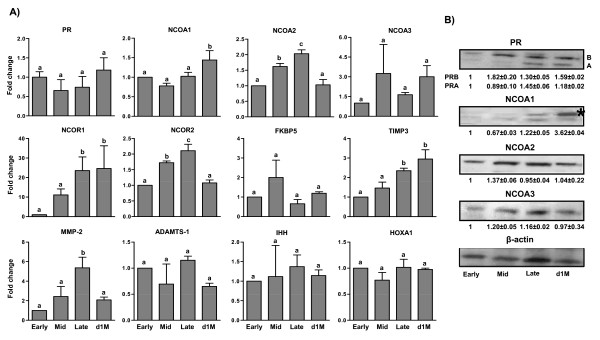
**Analysis of expression of genes encoding PR, its cofactors and P_4 _target genes in CL during different stages of the luteal phase in monkeys**. A) Real time RT-PCR fold change in expression (represented as mean ± SEM; n = 3 animals/stage of the luteal phase) of various genes associated with PR signaling and its target genes. The fold expression change at early stage was set as 1 and fold changes in other stages were expressed in relation to the early stage. Bars with different letters indicate significant (P < 0.05) change in the expression of the individual gene examined. B) Immunoblot analysis of PR, NCOA1, NCOA2 and NCOA3 expression in CL collected during various stages of the luteal phase. Immunoblots were analyzed by densitometry, and the densitometric value of early stage CL for each protein was set as 1 and values at other stages of CL were expressed in relation to the early stage. For NCOA1 protein, the upper band indicated by asterisk was considered for densitometric analysis. The statistical analysis details for densitometric values for each protein are provided in the results section.

Real time RT-PCR analysis results of PR, NCOA1-3, NCOR1-2, FKBP5, TIMP 3, MMP-2, ADAMTS-1, IHH and HOXA1 in CL collected from different stages of luteal phase are represented in Figure 2A. In order to gain information on the fold expression changes during different stages, expression of each gene at early (E) stage was set as 1 and expression at other stages of luteal phase was represented as fold change in relation to the early stage. Corresponding to the waxing and waning levels of circulating P_4 _throughout different stages of the luteal phase, the expression of PR mRNA was not statistically different (P > 0.05) across all the stages of luteal phase (Figure 2A). The protein expression for PR isoform B was low at early stage and its expression was high at all the other stages examined, with higher expression at mid luteal phase compared to early stage (1 vs 1.82 ± 0.2 fold; early vs mid stage, respectively; P < 0.05) (Figure 2B). However, the protein expression for PR isoform A was low at early and mid stage, and became higher at later stages of the luteal phase with late luteal stage being significantly higher (1 vs 1.45 ± 0.06 fold, early vs late stage, respectively; P < 0.05). The fold change in expression of NCOA1 mRNA and protein was low at early and mid stage (Figure 2A), and was significantly higher (P < 0.05) in CL collected from monkeys on day 1 of menses (1 vs 3.62 ± 0.04 fold, early stage vs d1 M, respectively; P < 0.05). Expression of NCOA2 and NCOR2 mRNA was higher (P < 0.05) in the late stage CL compared to their expression at early, mid and d1 M stages (Figure 2A), and the protein expression for NCOA2 was high in mid stage CL. Expression changes of FKBP5 and NCOA3 mRNA did not vary significantly during different stages of luteal phase (P > 0.05; Figure 2A). The protein expression of NCOA3 was high in mid stage CL compared to early stage CL (1 vs 1.2 ± 0.05 fold, early vs mid stage, respectively; P < 0.05, Figure 2B). The fold change in expression of NCOR1 showed a progressive increase with expression being higher in CL of late stage and d1 M compared to other stages (Figure 2A). The expression of proteinase family genes such as TIMP3 and MMP-2 were higher in CL at late luteal stage compared to early and mid luteal stages. Expression of ADAMTS-1, IHH and HOXA1 in CL tissues collected at different stages of luteal phase did not vary significantly (P > 0.05; Figure 2A).

### Effects of P_4 _replacement on expression of PR, coactivators and corepressors during induced luteolysis

**Figure 3:**
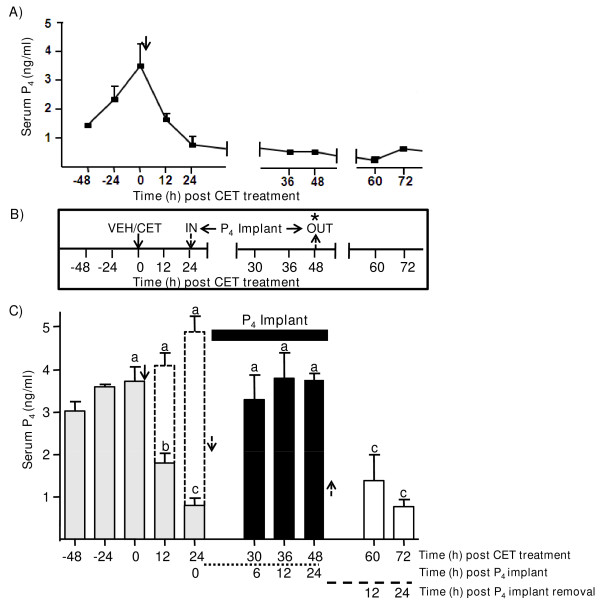
**Determination of luteal function after LH/P_4 _depletion and P_4 _replacement treatments in the monkey**. A) Circulating P_4 _levels before and after LH/P_4 _depletion [post CET administration (150 μg/kg BW) indicated by arrow; n = 3 animals] on day 7 of the luteal phase. P_4 _levels are represented only up to 72 h for purposes of comparing the levels with P_4 _replacement experiment [see panel C below]. Panel B depicts experimental protocol for VEH (5.25% glucose), LH/P_4 _depletion [CET (150 μg/kg BW)] and P_4 _replacement (CET+P_4_). The duration of P_4 _treatment initiated 24 h post LH/P_4 _depletion (n = 3 animals) in the form of P_4 _filled ALZET pumps is indicated by 'in' and 'out' words and arrows. The retrieval of CL after treatments is indicated by asterisk. C) Effects of VEH treatment (n = 3 animals), LH/P_4 _depletion (CET) (n = 3 animals) and [CET+P_4 _(n = 3 animals)] on circulating serum P_4 _levels. Data are presented as mean ± SEM. Before initiation of treatments, P_4 _values for each time point were pooled and represented as shaded bars (bars with dotted lines represent P_4 _concentrations after vehicle treatment). Following LH/P_4 _depletion and P_4 _replacement, P_4 _values are shown as open bars and solid bars, respectively. Serum P_4 _levels at 12 and 24 h after P_4 _implant withdrawal and CL removal are shown as open bars. Bars with different letters are significantly (P < 0.05) different.

Circulating P_4 _concentrations were 3.8 ± 0.3, 1.5 ± 0.07, 0.6 ± 0.1, 0.56 ± 0.01, 0.5 ± 0.04, 0.3 ± 0.05 and 0.6 ± 0.1 ng/ml, respectively at 0, 12, 24, 36, 48, 60 and 72 h post LH/P_4 _depletion (CET treatment; Figure 3A). All monkeys showed menses 96-120 h post LH/P_4 _depletion. A brief outline of the experiment involving exogenous P_4 _replacement is presented in Figure 3B. The P_4 _concentration in monkeys combined from all three groups was 3.5 ± 0.6 ng/ml prior to VEH (n = 3) or CET (n = 7) treatment (Figure 3C), and were maintained higher (4.1 ± 0.3 and 4.98 ± 0.3 ng/ml) at 12 and 24 h after administration of VEH, whereas P_4 _concentrations declined significantly 12 and 24 h post LH/P_4 _depletion (1.5 ± 0.07 and 0.6 ± 0.1 ng/ml at 12 and 24 h respectively; P < 0.05, Figure 3C). Replacement of P_4 _by way of implantation of P_4 _containing ALZET pumps at 24 h post CET treatment resulted in significant (P < 0.05) increase in P_4 _concentrations within 6 h post treatment and remained high throughout the 24 h duration of implantation. Upon removal of P_4 _containing ALZET pumps and lutectomy, P_4 _concentrations declined to reach 1.38 ± 0.6 and 0.76 ± 0.17 ng/ml at 12 and 24 h, respectively (Figure 3C).

**Figure 4:**
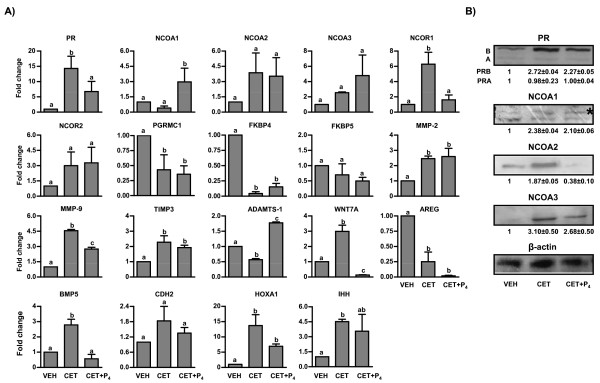
**Analysis of expression of genes associated with PR, its cofactors and P_4 _target genes in CL of monkeys treated with VEH (5.25% glucose injection at mid luteal phase), CET (LH/P_4 _depletion) and CET+P_4 _(P_4 _replacement)**. A) Real time RT-PCR expression analysis of various genes associated with PR signaling and its target genes. The fold expression change for VEH group was set as 1 and values for other groups were expressed in relation to the VEH group. For details on fold change calculation for real time RT-PCR analysis, see legend to Figure 1. Bars with different letters are significantly (P < 0.05) different for the individual genes examined. B) Immunoblot analysis of PR, NCOA1, NCOA2 and NCOA3 proteins in CL after LH/P_4 _depletion and P_4 _replacement. Immunoblots are analyzed by densitometry and densitometric value for VEH group is set as 1 fold and values for other groups were expressed in relation to the VEH group. For NCOA1 protein, the upper band indicated by asterisk was considered for densitometric analysis.

To analyze gene expression changes post P_4 _replacement, 19 genes were selected for real time RT-PCR analyses and the results were presented in Figure 4A. These genes were selected for analysis based on literature data from studies on classic P_4 _target organs, P_4 _responsive cell lines as well as from our microarray data of the LH/P_4 _depletion (CET induced luteolysis) experiments [16,28,29]. Initially the fold change in expression of PR mRNA was low in VEH treated monkeys, increased to 15 fold in LH/P_4 _depletion model, and then decreased significantly (P < 0.05) after P_4 _replacement (Figure 4A) suggesting PR to be a P_4 _target gene. Similar to pattern of PR mRNA expression, the protein expression of PR isoform B was low initially in CL of VEH treated monkeys and increased post LH/P_4 _depletion (1 vs 2.72 ± 0.04 fold VEH vs CET, respectively; P < 0.05; Figure 4B), while reduced post P_4 _replacement. However, the protein expression of PR isoform A was low in CL of VEH treated monkeys and remained low thereafter in both LH/P_4 _depletion and P_4 _replacement conditions (Figure 4B). The fold change in expression of PGRMC1 and FKBP4 mRNA was significantly lower (P < 0.05) after LH/P_4 _depletion and no significant effect was observed after P_4 _replacement (Figure 4A). Expression of NCOA1 mRNA decreased marginally after LH/P_4 _depletion while P_4 _replacement increased its expression significantly by 3-4 fold (P < 0.05; Figure 4A) suggesting NCOA1 mRNA to be regulated by P_4 _directly. However, protein expression for NCOA1 increased significantly after LH/P_4 _depletion (1 vs 2.38 ± 0.04 fold, VEH vs CET treatment; P < 0.05) (Figure 4B), but no significant change was observed following P_4 _replacement (P > 0.05). Messenger levels of NCOA2 and FKBP5 in CL tissue did not significantly change after LH/P_4 _depletion or P_4 _replacement (P > 0.05; Figure 4A). On the other hand, the protein expression for NCOA2 was significantly (P < 0.05) higher after LH/P_4 _depletion (1 vs 1.87 ± 0.05 fold, VEH vs CET treatment; P < 0.05). However, P_4 _replacement resulted in significant decrease in protein levels (1 vs 0.38 ± 0.10 fold, VEH vs CET+P_4 _treatment; P < 0.05) compared to CL obtained from VEH or CET treated monkeys indicating indirect/direct regulation of NCOA2 by P_4 _at the protein level. Messenger levels of NCOA3 in CL tissue did not change significantly after LH/P_4 _depletion or P_4 _replacement (P > 0.05; Figure 4A). However, the protein expression for NCOA3 was low in CL from VEH treated monkeys and increased significantly after LH/P_4 _depletion (1 vs 3.10 ± 0.50 fold, VEH vs CET treatment; P < 0.05) while decreased after P_4 _replacement (Figure 4B) suggesting a role for P_4 _in the regulation of NCOA3 protein levels. The fold change in expression of NCOR1 mRNA increased significantly 5-8 fold after LH/P_4 _depletion and became lower following P_4 _replacement (P < 0.05; Figure 4A). Similarly expression of NCOR2 mRNA increased (but not statistically significant) after LH/P_4 _depletion, but had no effect following P_4 _replacement (Figure 4A). Expression of WNT7A mRNA increased significantly (P < 0. 05; Figure 4A) after LH/P_4 _depletion, and the expression decreased significantly (P < 0.05) after P_4 _replacement demonstrating regulation of WNT7A mRNA by P_4_. Expression of AREG mRNA decreased significantly (P < 0.05) after LH/P_4 _depletion and remained low post P_4 _replacement (Figure 4A). The fold change in expression of BMP5 increased 3 fold after LH/P_4 _depletion, but the expression decreased significantly (P < 0.05) upon P_4 _replacement (Figure 4A) suggesting BMP5 gene to be a P_4 _responsive gene. Though the fold change in expression of cadherin (CDH2) tended to be higher (but not statistically significant) after LH/P_4 _depletion showed no significant change after P_4 _replacement (Figure 4A). Expression of HOXA1 and IHH mRNA were increased significantly (P < 0.05) after LH/P_4 _depletion, while expression of HOXA1 mRNA decreased after P_4 _replacement, expression of IHH mRNA did not change significantly after P_4 _replacement (Figure 4A).

The fold expression change of ADAMTS-1 mRNA after LH/P_4 _depletion was lower compared to VEH treatment (P < 0.05) and the expression increased significantly (P < 0.05) following P_4 _replacement (Figure 4A). Fold change in expression of MMP-9 mRNA increased post LH/P_4 _depletion, and decreased significantly post P_4 _replacement (P < 0.05; Figure 4A) indicating indirect/direct regulation of both ADAMTS-1 and MMP-9 mRNAs by P_4 _at the transcription level. Further, the fold change in expression of other genes like MMP-2 and TIMP3 increased significantly following LH/P_4 _depletion, but P_4 _replacement had no effect (P < 0.05; Figure 4A).

**Figure 5:**
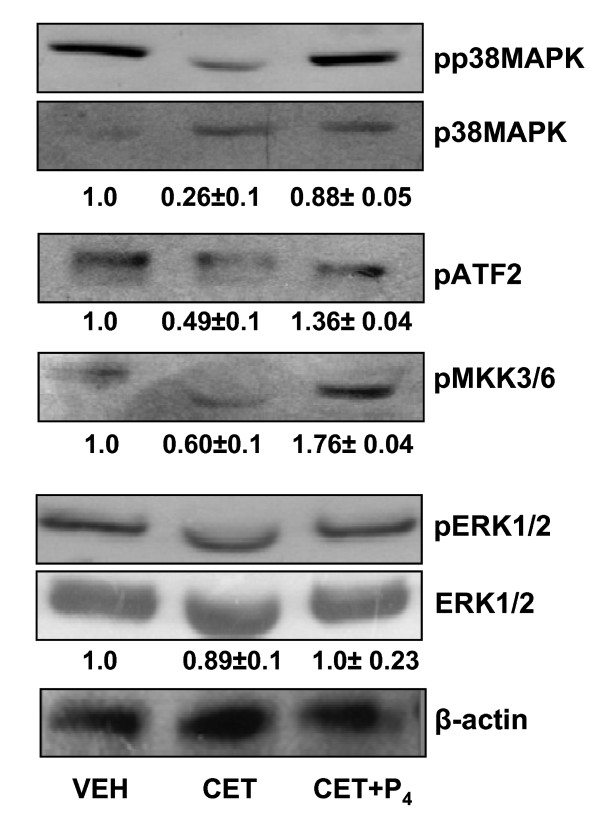
**Changes in activation levels of MAPKs during LH/P_4 _depletion and P_4 _replacement treatments**. Tissue lysates were prepared from CL of monkeys receiving VEH, CET (LH/P_4 _depletion) and CET+P_4 _(P_4 _replacement) treatments. The immunoblots shown are from one of three independent experiments (CL from one monkey at each time point was used per experiment). Immunoblots for phospho MAPKs and total MAPKs were analyzed by densitometry and the fold change in the protein levels of phospho/total MAPKs compared between treatments are represented numerically. β-actin was used as loading control.

Analysis of luteal tissue lysates for changes in P38 activation indicated that P38 activity was lower in CL after LH/P_4 _depletion, however, P_4 _replacement increased P38 activation significantly (P < 0.05; Figure 5). In vitro MAPK assays revealed lower levels of pATF-2, a substrate for activated P38 kinase, in CL after LH/P_4 _depletion, whereas, the effect of LH/P_4 _depletion on pATF-2 levels was prevented by P_4 _replacement (Figure 5). The kinetics of P38 activation was correlated with activation of its upstream kinase, pMKK3/6 protein levels. LH/P_4 _depletion decreased the pMKK3/6 protein expression significantly (P < 0.05), while P_4 _replacement increased its levels significantly (P < 0.05; Figure 5) indicating other components of MAP kinase signaling to be active post P_4 _replacement. Expression of other MAP kinase like ERK was also determined. Phosphorylated ERK levels did not change significantly in CL tissues both after LH/P_4 _depletion and P_4 _replacement (P > 0.05; Figure 5).

### Effects of P_4 _supplementation on gene expression changes during the late luteal phase

**Figure 6:**
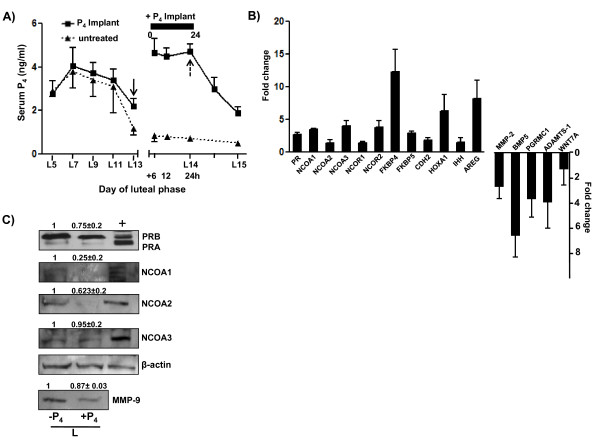
**Effects of P_4 _supplementation on luteal function during the late luteal phase**. Panel A, circulating P_4 _levels during P_4 _supplementation (n = 3 animals) with exogenous treatment by way of ALZET pumps for 24 h beginning on day 13 of the luteal phase to mimic high P_4 _levels similar to mid luteal phase. Panel B, real time RT-PCR expression analysis (mean ± SEM) of various genes associated with PR signaling and PR activated target genes are represented. Panel C, immunoblot analysis for PR, NCOA1-3 and MMP-9 proteins late luteal phase CL after P_4 _supplementation. Monkey term placenta tissue lysate was used as positive control for immunoblot analysis. The blots are analyzed by densitometry and densitometric values for untreated control group were set as 1 and values in P_4 _supplemented group were expressed in relation to the control group. The immunoblots shown are from one of three independent experiments (CL from one monkey at each time point was used per experiment). β-actin was used as loading control.

Circulating P_4 _concentrations in monkeys before and after implantation of P_4 _containing ALZET pumps and in untreated control monkeys are shown in Figure 6A. Implantation of P_4 _pumps resulted in significant (P < 0.05) increase in concentrations of P_4 _i.e., 4.4 ± 0.64, 4.2 ± 0.37, 4.4 ± 0.35 ng/ml at 6, 12, 24 h post P_4 _supplementation, respectively and the corresponding P_4 _concentrations from untreated control monkeys were 0.70 ± 0.1, 0.6 ± 0.1 and 0.49 ± 0.03 ng/ml at 6, 12 and 24 h, respectively (Figure 6A). Real time RT-PCR analysis showed >2 fold up regulation of PR, NCOA1, NCOA3, NCOR2, FKBP4, FKBP5, CDH2, HOXA1 and AREG expression in P_4 _supplemented monkeys, while expression of BMP5, MMP-2, PGRMC1, and ADAMTS-1 was significantly down regulated (P < 0.05; Figure 6B). Immunoblot analysis for PR (both isoforms A&B) protein showed no significant change after P_4 _supplementation, whereas, immunoblot analysis for NCOA1 and NCOA2 proteins showed significantly lower (P < 0.05) expression after P_4 _supplementation (NCOA1, 1 vs 0.25 ± 0.2 fold, for NCOA2; 1 vs 0.62 ± 0.2 fold; Figure 6C). Immunoblot analysis for protein expression of NCOA3 and MMP-9 showed lower levels after P_4 _supplementation (not statistically significant P > 0.05; Figure 6C).

## Discussion

Together with earlier reports for presence of PR in CL of many other species, the results of expression of PR, various binding proteins, cofactors and P_4 _regulation of expression of few genes in the present study further confirm P_4 _actions within the CL. The importance of P_4 _in the regulation of CL structure and function has been demonstrated in rats. It was shown that P_4 _administration during postpartum period had an anti apoptotic action in the CL [9]. Although the rat CL does not express nuclear PR, it was suggested that P_4 _mediates its action by binding to its membrane receptors [30]. In recent years, the membrane receptor-mediated actions of P_4 _appear to have emerged as an important mechanism for activation of P_4 _signal transduction pathway. It was reported that rat luteal cells express membrane and progestin binding proteins, progestin membrane receptor (PMR) α, PMR β, PMR γ, membrane component 1 (PRMC1/PGMRC1) and Rda 288 [30]. In a recent study, it was demonstrated that PGMRC1-dependent mechanism appears to promote human granulose/luteal cell survival [31] confirming the novel membrane-bound progestin receptors with defined actions. In the present study, expression of PGRMC1 was demonstrated in the CL and its expression became lower after LH/P_4 _depletion. This finding was similar to the earlier observation reported previously in the rhesus macaque [6]. Further restoration of PGRMC1 expression was observed after LH replacement in LH/P_4 _ablated rhesus macaque [6]. In contrast, in the present study PGRMC1 expression was not restored following LH replacement in LH/P_4 _depleted monkeys. The difference in the findings between the two studies could be attributed to differences in the treatment protocols employed and/or differences in the response to LH replacement observed between the two macaques.

Rothchild [2] aptly described CL as the most unique endocrine gland in the body, since there appears to be large interspecies variations in the mechanisms of regulation of its function. In higher primates, it is well established that the sole trophic stimulus for P_4 _secretion is the pulsatile secretion of pituitary LH. It is possible that P_4 _may not have an important role in its own secretion, but it might have an important role in maintaining structural integrity of the CL capable of responding to the luteotrophic stimulus. Hutchison and Zeleznik [32] demonstrated the rescue of CL function in the hypothalamus lesioned monkeys after reestablishment of pulsatile GnRH treatment indicating the resilience of CL tissue to the deprivation of luteotrophic support for long periods. In our studies, it was confirmed that CL was responsive to luteotrophic support for up to 48 h following inhibition of pituitary LH secretion [16]. We utilized LH/P_4 _depleted and LH replacement model systems that did not require extreme surgical procedures, yet allow examining the CL function during LH/P_4 _depletion period as well as hormone replacement period. During induced luteolysis, even though LH replacement had profound effects on expression of several genes [16], but this could be the result of both direct as well as indirect effects of LH. In the present study, examination of direct effects of P_4 _on CL tissue revealed changes in expression of PR and NCOR1, but expression of many other genes involved in PR activation were not significantly affected. Since, the duration of P_4 _replacement lasted only for 24 h, for the effect to be apparent a longer duration of P_4 _replacement might be required. Alternatively, perhaps the effects of P_4 _on expression of genes may be observed only when high intraluteal P_4 _levels as seen in functional CL is achieved. In the present study, only circulating P_4 _levels were mimicked but more studies are required to achieve the high intra luteal P_4 _levels and determine its effect on the luteal expression of genes.

Tissue proteinases and genes associated with tissue remodelling namely, TIMPs, MMPs and ADAMTS-1 have been examined in the CL tissues of higher primates. In the present study, the findings of higher expression of TIMP3 and MMP-9 was observed during latter part of the luteal phase which is in accordance with similar findings reported for CL of the rhesus macaque [18]. It was previously reported that ADAMTS-1 expression was high in early CL but declined thereafter at other stages of the luteal phase in the rhesus macaque [22]. In the present study, however, no discernible pattern of expression of ADAMTS-1 was observed during the luteal phase. It should be pointed out that in the rhesus macaque the highest expression was seen only at day 2 of the luteal phase, while in the present study the day 2 CL was not examined. Treatment with GnRH R antagonists [i.e. LH/P_4 _depletion models] in both the macaques resulted in decreased expression of ADAMTS-1 at mid luteal phase. Interestingly, LH replacement, but not steroid replacement, prevented the decreased ADAMTS-1 expression seen after GnRH R antagonist treatment in the rhesus macaque, but in the present study both LH (observed only in real time RT-PCR analysis) and P_4 _replacement treatments restored or increased its expression. The reason for different findings on ADAMTS-1 expression after P_4 _(in the present study) or progestin (rhesus monkey; [22]) treatments is difficult to explain and perhaps, related to treatment protocol employed in both these studies. Also, the unexpected decrease in ADAMTS-1 expression seen after P_4 _supplementation at late luteal phase observed in the present study is difficult to explain. Additional experiments are necessary to address the regulation of ADAMTS-1 expression in the CL by LH and P_4_.

Stouffer and Young, 2004 [19] reported a differential expression of MMP-9 with regulation of its expression to be at different levels involving both transcriptional and translational mechanisms in the CL of the rhesus macaque. In the present study, expression of MMP-9 in LH/P_4 _depletion and LH replacement studies remained high, but P_4 _replacement decreased its expression, suggesting MMP-9 expression appear to be regulated by P_4_. In accordance with our data, regulation of MMP-9 expression by P_4 _had been reported by other investigations in studies involving human endometrial explants and rabbit cervix [33,34]. It should be pointed out that MMP-9 expression during spontaneous luteolysis [data not shown] and P_4 _supplementation during late luteal phases did not show significant change indicating that regulation of MMP-9 expression may be dependent on circulating LH as well as P_4 _concentrations. The decrease in expression of MMP-2 mRNA post P_4 _replacement during late luteal phase was similar to the reported decrease in MMP-2 expression in the human CL after treatment with hCG to mimic early pregnancy [20]. It is possible that LH and P_4 _may synergistically act to regulate MMP-2 mRNA expression during the late luteal phase following P_4 _replacement as both are present in this model system, however, the molecular mechanisms for this synergistic action needs to be clarified. Decreased expression of pro-MMP-9, pro-MMP-2 and MMP-2 in endometrial cancer cell lines after in vitro administration of medroxy progesterone acetate, an synthetic progesterone preparation, [35] suggests that ECM remodelling could well be controlled by P_4 _as well as by LH in the CL tissue.

Expression of WNT7A, AREG, BMP5, HOXA1 and IHH, which are regarded as P_4 _target genes in uterus and mammary gland were examined in the CL after LH/P_4 _depletion and P_4 _replacement. WNT7A coordinates a variety of cell and developmental pathways and reported to guide hormonal responses during postnatal uterine growth [36]. In the present study, the observation that P_4 _decreased WNT7A expression was similar to finding reported in the uterus [25]. The observation of down regulated expression of BMP5 upon P_4 _replacement in the present study is consistent with the findings in uterus. Both WNT7A and BMP5 pathways appear to be interlinked since BMPs are shown to be induced by WNT signaling in the neurons [37]. BMPs have been shown to stimulate steroidogenesis and proliferation in porcine theca cells [38], but the role of P_4 _regulated WNT and BMP genes on the structure and function of CL remains to be determined. The observation of low levels of phosphorylated P38 in CL post LH/P_4 _depletion, and its reversal after P_4 _replacement are consistent with similar findings reported in uterus and mammary gland after P_4 _treatment in mice [39]. By activating the MAP kinase pathway, P_4 _appears to activate a unique biologically important molecule to control CL structure and function. It should be pointed out that changes in dynamics of MAP kinase activities have been shown to regulate CL function [27]. PR is known to be phosphorylated by various kinases including MAP kinases in response to various physiological stimuli [40]. The activation of MAP kinases have been shown to regulate expression and function of coactivators like NCOA1 [39] and NCOA3 [41]. Ligand-mediated down regulation and extensive loss of nuclear steroid receptor protein is well recognized and during this process, different phosphorylation pathways may affect the proteasome-mediated degradation of nuclear receptors including PR [40]. It is reported that NCOA3 is phosphorylated by P38 and the phosphorylated NCOA3 is subjected to degradation by proteasome [42]. Whether activation of P38 by P_4 _would have a role in regulating similar processes in the CL tissue remains to be addressed, however, the results in the present study suggest operation of similar mechanism. Gene expression analysis of 19 genes belonging to PR signaling during LH/P_4 _depletion and P_4 _replacement experiments revealed expression of 8 genes [(PR, NCOA1, NCOR1, WNT7A, BMP5, HOXA1, ADAMTS-1 and MMP-9) and protein expression of 3 genes] to be directly regulated by P_4_. The expression of other 11 genes appear to be regulated by both LH and P_4 _in the CL (see Table 3). In order to further extend the observations of direct actions of P_4 _and to further delineate role of P_4 _in the regulation of CL function, circulating P_4 _concentrations were increased for 24 h to mimic early pregnancy levels during the late luteal phase of the non fertile cycle. Expression of many of the genes examined was observed to be differentially expressed indicating that P_4 _may have an important role in the regulation of CL function during early pregnancy. Also, it should be pointed out that the general housekeeping activity that include anti-apoptotic actions, structural integrity of luteal cells and perhaps regulation of expression of few genes associated with overall maintenance of CL structure may be attributed to direct or indirect actions of P_4_.

**Table 3:** List of genes whose expression identified to be regulated by P_4 _treatment

**Sl. No**.	Gene		Changes in levels after		Regulation by P_4_
			LH/P_4 _depletion	P_4 _replacement	
1	PR	mRNA	up	down	Y
		Protein (PR B)	up	down	Y
		Protein (PR A)	-	-	N
2	NCOA1	mRNA	-	up	N
		Protein	up	-	N
3	NCOA2	mRNA	-	-	N
		Protein	up	down	Y
4	NCOA3	mRNA	-	-	N
		Protein	up	down	Y
5	PGRMC1		down	-	N
6	FKBP4		down	-	N
7	FKBP5		-	-	N
8	NCOR1		up	down	Y
9	NCOR2		up	-	N
10	WNT7A		up	down	Y
11	AREG		down	down	N
12	BMP5		up	down	Y
13	CDH2		up	-	N
14	HOXA1		up	down	Y
15	IHH		up	-	N
16	ADAMTS1		down	up	Y
17	MMP9		up	down	Y
18	MMP2		up	-	N
19	TIMP3		up	-	N

In summary, experiments were performed to examine direct effects of P_4← _on the regulation of expression of genes in the monkey CL. The first experiment comprised of analysis of previously published microarray data to identify differentially expressed genes considered as target of P_4 _action and validation of some of these genes by real time RT-PCR analysis. In the second experiment, expression of many of the genes identified in the first experiment was examined throughout the luteal phase. In the third experiment, the direct effects of P_4← _on expression of many of these genes was examined following P_4← _replacement in monkeys depleted for circulating endogenous LH and P_4← _by way of inhibition of pituitary gonadotropin secretion. In the fourth experiment, the effects of P_4← _supplementation on expression of genes during the late luteal phase with declining endogenous P_4← _levels were examined. This experiment was done to determine whether increased P_4← _seen during early pregnancy regulate expression of these genes. The results from these studies indicated that P_4← _appears to regulate expression of many of the genes in the CL.

## Conclusions

Experiments were carried out to assess the direct effects of P_4 _on CL function in monkeys. Expression of some of the genes involved in PR signaling and genes considered as targets of LH and P_4 _was analyzed in LH/P_4 _depletion and P_4 _replacement model as well as P_4 _supplementation model during the late luteal phase, and the results indicated that expression of number of genes appeared to be regulated directly or indirectly by P_4_. These results suggest that replacement of P_4 _during LH/P_4 _depletion (induced luteolysis model) is suitable for assessing the effects of P_4 _on CL function. Further, these studies suggest that CL could serve as a target tissue for examining genomic and non genomic actions of P_4_.

## Competing interests

The authors declare that they have no competing interests.

## Authors' contributions

PS and RM participated in designing, conducting experiments, analysis of results and preparation of manuscript. KCJ participated in the preparation of manuscript. All authors read and approved the final manuscript.
